# Institutional Notes and News

**Published:** 1910-07-23

**Authors:** 


### Institutional Notes and News.

**Leeds Maternity Hospital.**

The Leeds Maternity Hospital can congratulate itself on having been recognised as one of the medical charities of the town, apart from the fact that this recognition means that the hospital will receive five instead of one per cent. from the Hospital Sunday Collection. The work done last year was considerable, 563 patients were treated, or more than three times as many as in 1908. This progressive demand on the hospital's resources seems likely to continue unabated, and the number of pupils applying for the training given is also on the increase. The accounts for the year ended with a surplus of £240, a much smaller one than that with which the year began, owing to the diversion of many subscriptions to the building fund. This fund requires £7,000 to enlarge the hospital, of which £5,000 has been subscribed, and now a further £5,000 is required for furnishing and other expenses. The report at the recent annual meeting was presented by Mrs. Robert Hudson.

**East End Mothers' Lying-in Home.**

The East End Mothers' Lying-in Home, Commercial Road, was fortunate in having such an excellent place and such excellent speakers for its annual meeting, which was presided over by the Lord Mayor at the Mansion House. He said he hoped that its appeal for funds would be responded to heartily, and was supported in this by the Bishop of Stepney, who acutely and eloquently remarked that the home was typical of the East End, for its peculiar characteristics were the tenderness, affection, and enthusiasm with which it was carried on. Last year a great amount of work was accomplished in both internal and external ways; for instance, 573 in-patients were treated and 854 out-patients. Besides this, however, more than 17,000 visits were made by the nurses of the home, for the double purpose of attending mothers in their own homes and of giving advice as to how young children should be taken care of. It has often been pointed out in these columns how important is the education of the mother if the children are to survive, and survive healthily; any lying-in home, therefore, which pays attention to the educative as well as to the medical side of its work is doing capital service to the State. The other speakers included Mrs. Quintin Hogg, Dr. Owen Lankester (chairman of the Home), and Lord Goschen.

**North Devon Infirmary.**

After having adopted the extreme measure of closing one of the wards, the governors of the North Devon Infirmary met the other day to revise the privileges given to subscribers. Mr. C. E. R. Chanter, chairman of the House Committee, summed up the state of affairs by saying that the falling off in the hospital's income was found under the extraordinary division of that income. The governors must either sell out some of the invested funds or curtail the "recommends." As it was necessary to keep the hospital up to date one could not reduce expenditure. The revision of privileges will mean that anyone who gives £25 to the institution will receive one recommendation instead of two and a-half. Previously, anyone who gave ten guineas, or an annual subscriber of one guinea, was allowed to recommend one in-patient or five out-patients. One indoor "recommend" admits a patient for three weeks. The general feeling appears to be that of the House Committee, voiced by Mr. Chanter, that a patient is kept too long in the hospital for the sum paid by subscribers. So, after a good deal of discussion, the following rule (26) was passed: "No in-patient shall remain in the infirmary longer than fourteen days on one recommendation, but, if it be considered necessary by the medical officer and approved by the House Committee, a patient may remain for such period necessary by one quarter of recommend for each four days the patient may remain." Mr. Dunn Pattison, vice-chairman, attributed the saving of £300 per annum on the provision bill to the careful management of Miss Lillie, the matron.

**Birmingham Saturday Fund.**

The Birmingham Hospital Saturday Fund is to be congratulated on the result of this year's annual collection, which has reached nearly £20,000, or £535 more than last year. Its Chairman at the recent meeting, Sir Hallewell Rogers, also announced that the plans which the Fund had been cherishing of having a hospital for consumptives were nearly complete. It has been proposed that this hospital should take the form of a memorial to Sir William Cook, who was Chairman of the Fund for many years. After more than one difficulty, a suitable site of 25 acres on the outskirts of Great Farley Wood, Romsley Hill, within ten miles of the city, has been found, and, what is more, has been given to the Fund. It is hoped to provide accommodation for fifty beds, and of the £12,000 required to build the Sanatorium here, some £5,000 has been received or promised. More than one person in Birmingham believes that this Home for Consumptives will not only be a blessing to the city but also add to the popularity of the Fund. The responsibility for maintaining the Home will lie, of course, on the Fund, and therefore it must aim, as Sir Hallewell Rogers pointed out, at increasing the yearly sum subscribed to £25,000.

**Middlesex Hospital.**

The important occasion of laying the foundation-stone of the new buildings for the research and treatment of cancer at the Middlesex Hospital secured the finest day of the week. Prince Francis of Teck, the Chairman of the Weekly Board, who has been very busy with the successful presentation of the appeal, performed the ceremony. The Queen, who was prevented from doing this office owing to the death of King Edward, has promised, however, to declare the buildings open next year, when they will be ready for use. It will be remembered that these buildings are the outcome of a fund left by the late Mr. Henry I. Barnato to build or endow a charitable institution in memory of his brother, Mr. Barnett I. Barnato, and of his nephew, Mr. Woolf Joel. The fund reached the splendid proportions of some £300,000, of which the new buildings are expected to appropriate £50,000. There remains, therefore, a magnificent endowment to maintain them, and to prevent them from becoming the white elephant which similar buildings have more than once elsewhere. The buildings themselves, of brick and Portland stone, are to rear their five stories opposite Nassau Street, west, that is, of the main hospital block. They will contain, in their main block and two wings respectively, two big wards, out-patients' department, and examination rooms, plus accommodation for the nurses. Of the wings, that on the north-east of the new department will contain the laboratories and apparatus for research, while the remaining new wing will be occupied by the nurses' home and an electrical department. It should be added that the existing cancer wing will be reconstructed also. After the formal opening Mr. S. B. Joel moved a vote of thanks to Prince Francis, and presented him with a silver-gilt casket containing the mallet and trowel previously used by him.

